# Initial hydraulic failure followed by late-stage carbon starvation leads to drought-induced death in the tree *Trema orientalis*

**DOI:** 10.1038/s42003-018-0256-7

**Published:** 2019-01-07

**Authors:** Yuri Kono, Atsushi Ishida, Shin-Taro Saiki, Kenichi Yoshimura, Masako Dannoura, Kenichi Yazaki, Fuku Kimura, Jin Yoshimura, Shin-ichi Aikawa

**Affiliations:** 1 Center for Ecological Research, Kyoto University, Otsu, Shiga 520-2113 Japan; 2 Forestry and Forest Products Research Institute, Tsukuba, Ibaraki 305-8687 Japan; 3 Faculty of Agriculture, Yamagata University, Tsuruoka, Yamagata 997-8555 Japan; 4 Kyoto University Graduate School of Global Environmental Studies, Kyoto, Kyoto 606-8502 Japan; 5 Faculty of Agriculture, Kyoto University, Kyoto, Kyoto 606-8502 Japan; 6 Graduate School of Bioresource Sciences, Nihon University, Fujisawa, Kanagawa 252-0880 Japan; 7 Graduate School of Science and Technology and Department of Mathematical and Systems Engineering, Shizuoka University, Naka-Ku, Hamamatsu Shizuoka, 432-8561 Japan; 8 Department of Environmental and Forest Biology, State University of New York College of Environmental Science and Forestry, Syracuse, NY 13210 USA; 9 Marine Biosystems Research Center, Chiba University, Kamogawa, Chiba 299-5502 Japan; 10 Japan Forest Technology Association, Chiyoda, Tokyo 102-5281 Japan; 11 Graduate School of Science and Engineering, Tokyo Metropolitan University, Minami-Osawa, Hachioji, Tokyo 192-0397 Japan

## Abstract

Drought-induced tree death has become a serious problem in global forest ecosystems. Two nonexclusive hypotheses, hydraulic failure and carbon starvation, have been proposed to explain tree die-offs. To clarify the mechanisms, we investigated the physiological processes of drought-induced tree death in saplings with contrasting Huber values (sapwood area/total leaf area). First, hydraulic failure and reduced respiration were found in the initial process of tree decline, and in the last stage carbon starvation led to tree death. The carbohydrate reserves at the stem bases, low in healthy trees, accumulated at the beginning of the declining process due to phloem transport failure, and then decreased just before dying. The concentrations of non-structural carbohydrates at the stem bases are a good indicator of tree damage. The physiological processes and carbon sink-source dynamics that occur during lethal drought provide important insights into the adaptive measures underlying forest die-offs under global warming conditions.

## Abstract (web summary)

Yuri Kono et al. show that drought-induced tree death is caused by initial hydraulic failure followed by carbon starvation. They find that the loss of the carbon sink-source balance in stem bases due to phloem transport failure is the key factor governing wilting, providing insight to developing adaptive measures to prevent forest die-offs under global warming conditions.

## Introduction

Drought is expected to increase in frequency and severity in many biomes because of global climate change. Over the past decade, the destruction of forest ecosystems caused by heat waves and prolonged drought has been widespread over multiple biomes^1–4^, which has resulted in severe damage to the large carbon sink and high biodiversity in forest ecosystems globally. To help prevent the destruction of forest ecosystems, the physiological mechanisms underlying drought-induced tree die-offs have recently been studied intensively^5,6^. However, sufficient information is not yet available to predict patterns of forest destruction^7,8^. Two major hypotheses have been proposed to explain tree mortality caused by drought. The hydraulic failure hypothesis postulates that tree die-offs are largely a result of dysfunctional water transport caused by xylem embolism^9^, and the carbon starvation hypothesis suggests that die-offs are caused by shortages of carbohydrate reserves resulting from a decline in photosynthesis^5,10^. In the literature, the hydraulic failure hypothesis has been largely supported by evidence from field studies of mature trees^6,9,11,12^, whereas the carbon starvation hypothesis has been supported by evidence from experimental studies of tree seedlings^13^. Furthermore, the reduced non-structural carbohydrates (i.e., carbon starvation) are more common for gymnosperms than for angiosperms^14^. However, why both hypotheses are supported in different cases remains unclear.

The hydraulic failure and carbon starvation hypotheses alone are likely to be too simple and nonexclusive to explain drought-induced mortality because of the closely related carbon–hydraulic interactions^5,15–18^. Successive droughts lead to cumulative physiological damage toward drought-induced tree death, resulting in exhaustion of stored resources and nonreversible loss of regenerating structures^8^. When trees suffer from drought, they usually shed leaves to reduce their transpiration area^19,20^, meaning that the Huber value (sapwood area divided by total leaf area) is a good indicator of cumulative water stress. Soil water potential changes seasonally and daily, because of temporal rainfall. Therefore, Huber values, rather than predawn leaf water potential, are the only detectable parameters for accumulated dehydration damage over the long term, especially in field-growing trees^21^. Furthermore, the Huber value or the level of defoliation has more advantage for forest managers and policy-makers to easily find the level of drought-induced damage in adult trees.

Such defoliation can contribute to the recovery of hydraulic functions caused by the reduced transpiration area of the whole plant, although it results in reduced carbon gain^22^. Within sapwood, starch is converted to soluble sugars with the progression of xylem embolism formation during prolonged drought, whereas soluble sugars return to starch as embolized vessels are refilled following rainfall or irrigation^18^. Soluble sugars will be used in many metabolic processes, such as osmotic regulation^23,24^, energy-driven refilling of embolized vessels under negative pressure^25–30^, fine-root production^31^ and the secondary growth of new vessels in stems for water uptake. However, if carbon transport in phloem is strongly disturbed, the effective use of soluble sugar is limited, leading to tree death^15^. When trees die, the depletion of the carbon pool is found in roots, rather than in the aboveground tissue, leading to root death^31^. Furthermore, when trees are exposed to a lethal drought, the respiration rates in the stems decrease because of damage to living cells (i.e., metabolic failure)^18,21^. The reduced mitochondrial respiration decreases the energy produced by H^+^-ATPase at the plasma membrane. Such metabolic failure should reduce the hydraulic permeability in cell membranes as a consequence of decreased aquaporin activity^32^, which results in phloem turgor loss^30,33,34^ and reduced phloem transport^31,35–37^. Although the physiologically complex pathway is predicted for drought-induced tree die-offs^19^, a greater understanding of tree wilting processes can assist forest managers and policy-makers in developing adaptive measures to protect forest die-offs under future global warming^8^.

Here, we investigated the growth and survival of 290 2-year-old saplings (without pathogenic damage) with contrasting Huber values in *Trema orientalis* (L.) Blume for 1 year in the Ogasawara Islands, Japan. The pioneer trees can set seeds from 3-year-old. Using ten saplings with contrasting Huber values, we examined the physiological processes when trees die. Furthermore, using three saplings with contrasting Huber values, we compared the phloem function with ^13^C (stable isotope of carbon) labelling experiment in the field. Our results demonstrate that both the hydraulic failure and the carbon starvation hypotheses are valid, although they appear to relate to different stages of tree die-offs. We found that the nature of drought-induced tree die-offs can be understood based on the loss of the carbon sink–source balance in stem bases.

## Results

### Drought events in the study site

**Fig. 1:**
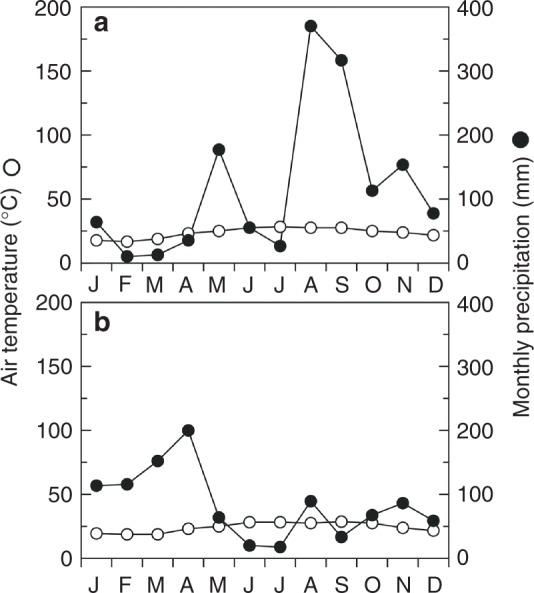
Heinrich Walter’s climate diagrams for monthly mean air temperature (open circles) and monthly precipitation (closed circles) in **a** 2015 and **b** 2016. Data are from the Japan Meteorological Agency

Conspicuous drought is usually detected in the islands, especially in the early summer. This study was carried out in 2015 and 2016, and drought was found in July in both years (Fig. 1). The soil water contents at the study site dropped extremely in July, when air temperature increased but the precipitation was relatively small (Supplementary Fig. 1).

### Relative growth rates decrease with increasing Huber values

**Fig. 2:**
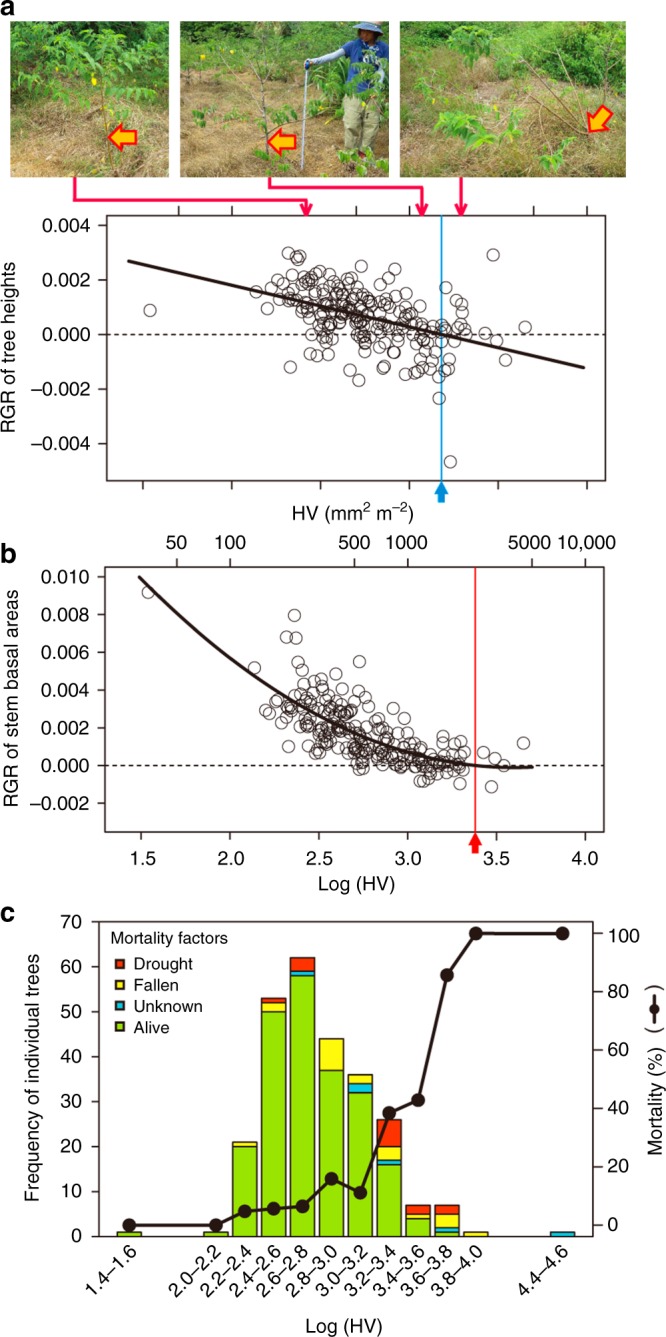
Relative growth rate (RGR) and the mortality over - year (June 2015–June 2016) in 290 2-year-old sun-exposed individual trees (pioneer *Treama orientalis*) with different Huber values (Huber values, sapwood area/total leaf area) in June 2015 (at 2 years old). **a** RGR of tree heights. **b** RGR of stem basal areas. **c** Vertical bars show the frequency of individual trees, tree survival (green), and causes of mortality (red: standing dead trees, yellow: fallen dead trees, blue: unknown). Note that standing dead trees (red) would almost drought-induced die-offs, and fallen dead trees (yellow) would include drought-induced die-offs. The polygonal line shows the variations in mortality along with different classes of (log-transformed) Huber values during 1 year (2015–2016). *X*-axes of **a** and **b** are the same scale. Closed circles in the panel **c** mean the mortality rates in each Huber value class

We examined the relationships between Huber values and plant growth among the 290 sun-exposed saplings. Relative growth rates (RGRs) over 1 year (from 2-year-old to 3-year-old) for the tree heights and stem cross-sectional area decreased and the tree-mortality rates increased as the Huber values increased (i.e., progression of defoliation) (Fig. 2). Because any pathogenic and herbivore damage were not detected, the main factor of tree death would be due to dehydration damage. These results indicate that the defoliation level is a good indicator of tree health. The fitting of the curve for the RGR of stem cross-sectional area against Huber values showed that the Huber value was 3.38 (mm^2^ m^−2^) when the RGR was 0 (Fig. 2b). However, a Huber value of 3.18 (mm^2^ m^−2^) was the threshold point for tree height growth (Fig. 2a). A margin occurred between the threshold points for height growth and stem diameter growth, and the sapling mortality increased rapidly within this margin (Fig. 2c). When the values of Huber value were more than 3.18 (mm^2^ m^−2^) in the summer of 2-years old, the mortality following 1 year reached 100%. According to the fact, a Huber value of 3.38 at 2-year-old would be the threshold of tree death, potentially the point of no return in this study.

### Hydraulic failure is an early stage of drought-induced death

**Fig. 3:**
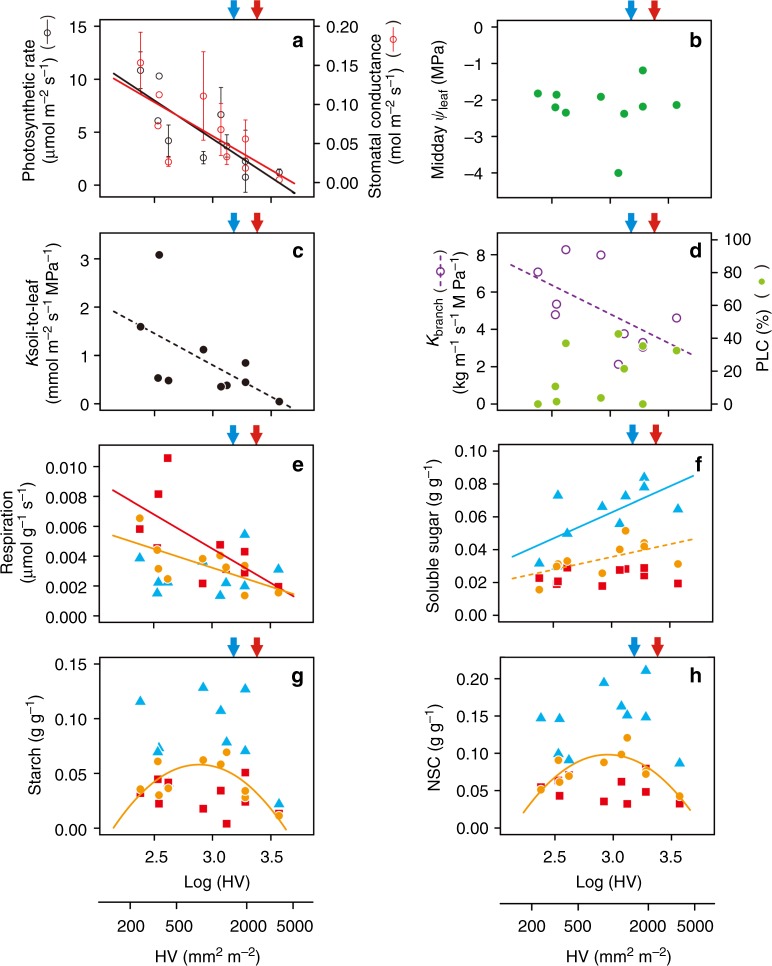
Physiological characteristics of ten individual two-year-old trees with different Huber values (Huber values). **a** Photosynthetic rate (black circles) and water-vapour stomatal conductance (red circles) (mean ± 1 SD). **b** Midday leaf water potential. **c** Soil-to-leaf hydraulic conductance (*K*_soil-to-leaf_). **d** Hydraulic conductivity (*K*_branch_: open circles with purple) and the percent loss of conductivity in healthy branches (PLC: closed circles with green). **e** Dark respiration rates in the healthy branches, stem bases and taproots (plant parts). **f** Soluble sugar concentrations within sapwood in the different plant parts. **g** Starch concentrations within sapwood in the different plant parts. **h** Concentrations of non-structural carbon (NSC: soluble sugars + starch) within sapwood in the different plant parts. **e**–**h** the values correspond to healthy branches (red squares), stem bases (orange circles) and taproots (blue triangles). The solid lines show significant correlations between the obtained values and log(Huber values) (*P* < 0.05), and the dashed lines show marginal correlations (0.05 ≤ *P* < 0.08). The blue and red arrows show the thresholds for tree height growth and stem diameter growth, respectively. The thresholds for stem diameter growth potentially corresponds to the point of no return (shown in Fig.1b, c)

We examined the physiological processes associated with tree decline by selecting ten individual trees with different Huber values (2.38–3.57 mm^2^ m^−2^) and similar stem diameters (19.8–24.9 mm at the stem base) without pathogenic and herbivore damage (Supplementary Fig. 2). Although we used the healthy branches in each tree, the leaf gas exchange rates decreased linearly with log(Huber value) (Fig. 3a). However, the midday leaf water potential remained constant (except for one tree), showing isohydric behaviour (Fig. 3b). The individual tree with the extremely lowest midday leaf water potential (Huber value = 3.07 mm^2^ m^−2^) was likely unable to compensate for excess water loss, even via stomatal closure and defoliation, which resulted in a temporary drop in leaf water potential. To restore (increase) its midday leaf water potential, this tree needs to increase defoliation. The soil-to-leaf hydraulic conductance (*K*_soil-to-leaf_) tended to decrease with the log(Huber value) (*P* = 0.0759) (Fig. 3c). Although a decreasing trend with respect to the hydraulic conductivity of healthy branches (*K*_branch_) was also found (*P* = 0.0794), the percentage loss of conductivity (PLC, an indicator of embolism in xylem vessels) remained unchanged in the healthy branches (Fig. 3d). The dark respiration rates (measured at 25 °C) also significantly decreased with the log(Huber value), even in the healthy branches and stem bases (*P* < 0.05) (Fig. 3e). Soluble sugars in the taproots increased significantly with the log(Huber value), and those in the stem bases increased marginally (Fig. 3f). Thus, respiration rates were not related to soluble sugar contents. In the stem bases, the NSC concentrations within the sapwood increased with the log(Huber value) and then decreased (Fig. 3g, h). Interestingly, the peak starch and NSC concentrations occurred before the thresholds for height growth and tree death. The dynamic change in NSC within sapwood was detected in only stem bases.

### Carbon starvation occurs latter in drought-induced death

**Fig. 4:**
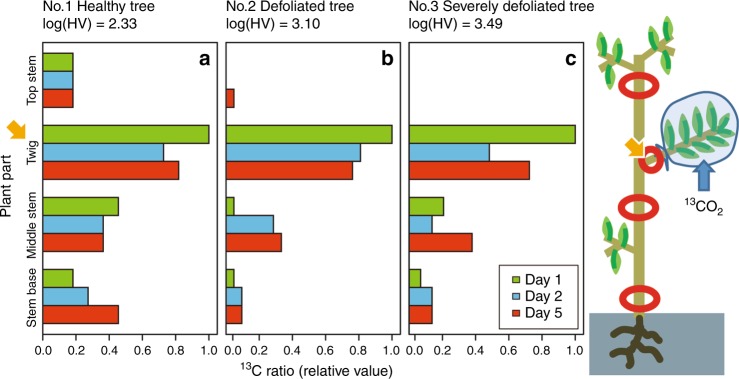
Carbon transport in phloem using a trace experiment with stable carbon isotope, ^13^C, labelling in the field. **a** ^13^C ratios in a healthy tree (log(Huber value) = 2.33), **b** in a defoliated tree (log(Huber value) = 3.10), and **c** in a severely defoliated tree (log(Huber value) = 3.49). The amount of ^13^C detected in the aboveground parts at 1 day (green bars), 2 days (blue bars) and 5 days (red bars) after labelling relative to the amounts of ^13^C in the labelled twig 1 day after labelling in each tree. The arrows show the labelled twig in each tree. In the severely defoliated tree, the top stem above the labelled twig has died back. The middle stem is the stem section between the labelled twig and the stem base. See the Methods for more details on this experiment

To examine whether the dynamics of NSC at the stem base are accompanied by the inhibition of phloem transport, we conducted a ^13^C (stable isotope of carbon) labelling experiment in the field using a healthy tree (Huber value = 2.33 mm^2^ m^−2^) and two defoliated trees (Huber values = 3.10 and 3.49 mm^2^ m^−2^) (Supplementary Fig. 2). Following the absorption of ^13^CO_2_ by healthy branches, the healthy tree showed a rapid upward and downward translocation of carbon one day later (Fig. 4a). Five days after labelling, the amount of carbon translocated into the stem bases was higher in the healthy tree than in the defoliated trees (Fig. 4c), thus indicating that the phloem transport capacity decreased with tree decline.

**Fig. 5:**
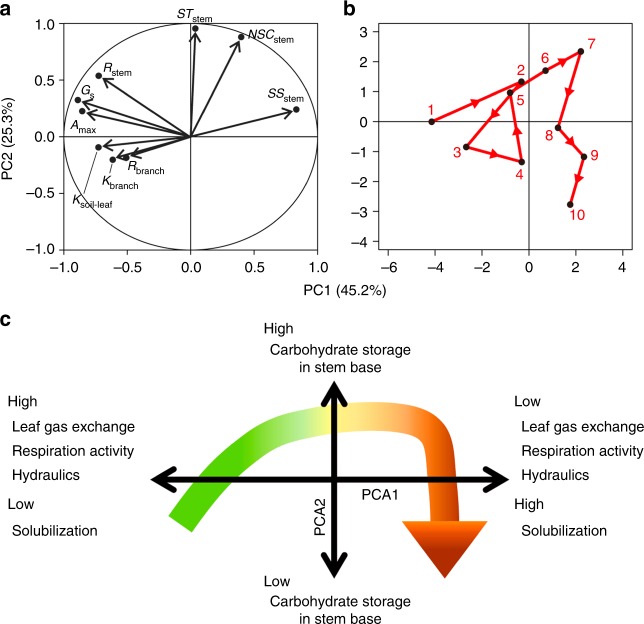
Principal component analysis (PCA) of ten individual trees with contrasting Huber values (Huber values). **a** Analysis of nine plant traits. **b** Results for the ten individual trees used in Fig. 2. The numbers in **b** indicate the order of increasing healthy trees to defoliated trees of increasing Huber values among the ten individual trees. **c** Results of the PCA. The arrow shows the physiological progression from healthy trees to wilting trees. Abbreviations are as follows: *A*: photosynthetic rates, *G*: the maximum stomatal conductance, *K*_branch_: hydraulic conductivity in healthy branches, *K*_soil-leaf_: soil-to-leaf hydraulic conductance, *R*_stem_: dark respiration rate in the stem bases, *R*_branch_: dark respiration rate in healthy branches, *ST*_stem_: starch in the sapwood of stem bases, *SS*_stem_: soluble sugar in the sapwood of stem bases and *NSC*_stem_: non-structural carbon in sapwood of the stem bases. Axes 1 and 2 explain 45.2% and 25.3% of the total variation, respectively

Using the obtained data (nine plant traits and ten individual trees in Fig. 2), the results of the principal component analysis (PCA) are shown in Fig. 5. Axes 1 and 2 explain 45.2% and 25.3% of the total variation, respectively. Axis 1 is related to the leaf gas exchange, respirational activity, hydraulic function (*K*_soil-to-leaf_) and solubilization from starch to sugars, and axis 2 is related to the NSC and starch concentrations in the stem bases. The healthy trees with low Huber values (i.e., leafy trees) exhibited a high level of physiological activity but relatively low carbohydrate reserves in the stem bases (near axis 1 on the left side of axis 2). With progressive defoliation (increasing Huber values), the photosynthesis, respiration rates and *K*_soil-to-leaf_ tended to decrease (towards the positive sides of both axes). Moreover, the NSC and starch concentrations in the stem bases increased and the phloem carbon transport was inhibited before the threshold for tree height growth was reached. With increasing defoliation towards the tree die-off threshold, the carbohydrate reserves in the stem bases decreased again (moving towards the negative side of axis 2 while remaining on the positive side of axis 1).

## Discussion

**Fig. 6:**
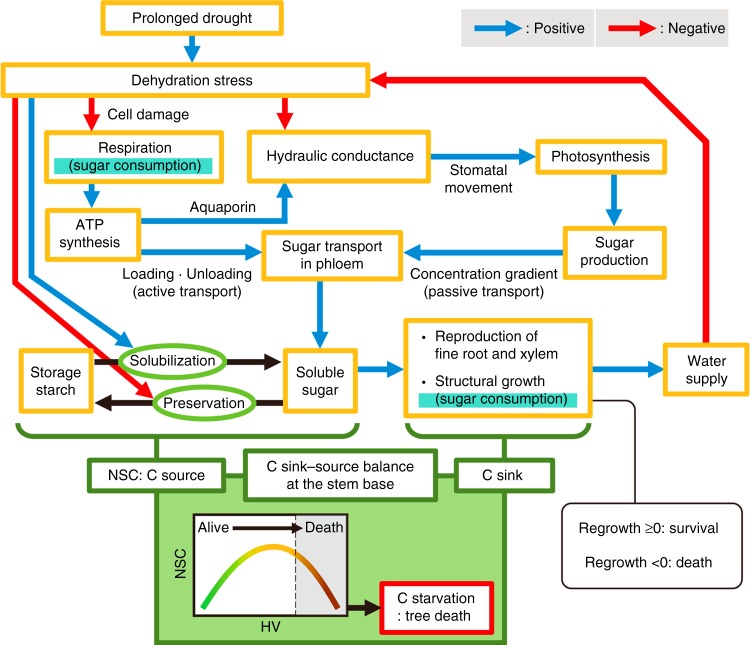
Schematic diagram of the tree die-off process. Blue arrows show positive effects, and red arrows show negative effects. See the main text for more details

This study has clarified the relationships between hydraulic failure and carbon starvation, leading to the drought-induced tree death. Based on the current results, we built a schematic diagram for drought-induced tree decline as a hypothetical framework (Fig. 6). In the initial stage of tree decline, respiration (cell metabolism) and leaf gas exchange decreased. Dehydration is known to damage living cells, such as the parenchyma and phloem, because of dehydration-induced shrinking^21,31,33^. Furthermore, dehydration decreases the water permeability in fine-root cell membranes^22^ and in out-xylem hydraulics in leaves^38^. Roots and leaves tend to be more vulnerable to dehydration than stems^39,40^. Hartman et al.^31^ showed that the phloem transport reduced under severe drought causes a decline in root systems by isolating them from the carbon source. Because pioneer trees generally have a high root-mass ratio^22^, the effects of declined root systems on whole-plant hydraulics will be severe. Therefore, the depression of leaf gas exchange likely would be connected with hydraulic failure in leaf hydraulics (*K*_leaf_) and root systems, rather than in xylem cavitation in branch. A reduction in hydraulic function decreases carbon production and results in a reduction in the passive transport of sugar in the phloem, because of a low sugar concentration gradient. Furthermore, a reduction in respiration (cell metabolism) would reduce the active transport of sugar in the phloem^33^. Dehydration promotes the solubilization of carbohydrate reserves within sapwood^18^ via osmoregulation and structural plant growth^23,24,31^. However, the impeded phloem function would disturb the effective use of solubilized sugar and would loss carbon sink–source balance at the stem bases. Because of the initial apparent increase and successive real decrease in stored carbon (including starch and NSC) at the stem base, the carbon sink–source balance at the stem base is a good indicator of tree health. Overall, this hypothetical framework in drought-induced tree die-offs suggests that hydraulic failure and metabolic failure seem to be in the initial stages, and NSCs are gradually consumed through these processes. Finally, carbon starvation occurs in the last phase of tree death.

Because the complete depletion of carbon reserves is rarely observed, tree death from carbon starvation has been often debated^41^. However, the reduced carbohydrate reserves (NSCs) have been frequently found in drought-induced dehydration damaged trees, especially in the stem base. Just before dying, the remaining NSCs would be an unusable carbon source, resulting from cell dehydration and damage^21^. Our data clearly show that reduced NSCs fairly occurred just before tree death, but the simple measurement of NSCs is not always the detectable method for the dehydration damage of trees. The healthy trees with high RGR and the most damaged trees with low RGR have low NSC, and interestingly the medium-damaged trees show the highest NSCs (i.e., extensive carbon accumulation). Phloem transport of NSCs is an essential factor for maintaining hydraulic function and structural plant growth during dehydration stress. However, phloem transport failure has rarely been experimentally investigated in studies on tree mortality^33^. Because the regrowth would require much soluble sugar^31^, the low phloem transport would impede plant regrowth and the recovery of water supply after dehydration damage. The phloem transport would be suppressed not only by low carbon gain but also by drought-induced cell damage, such as phloem turgor collapse^33^ and cell dehydration^21^. Therefore, the success/failure of regrowth will be the essential factor for determining the tree survival, and the NSCs dynamics at the stem bases along the accumulated dehydration damage will be due to the results of carbon source and sink balance. The phloem failure is obviously linked to hydraulic failure and carbon starvation. The seasonality of phloem transport has been described in winter-deciduous and over-wintering evergreen trees in cool-temperate zones^35^. However, in the current study, the effects of seasonality were not considered, because the studied woody plant (*Trema orientalis*) is a typically tropical pioneer tree in Asia and has no seasonal resting periods for plant growth and leaf emergence. This species is an evergreen tree, but its leaf lifespan is short with thin lamina, and defoliation is easily promoted under unfavourable environments^20,42,43^. Therefore, the disturbance of phloem transport shown in this study is likely to be caused by drought-induced tree damage, rather than seasonal change, dormancy, or ontogenetic variations.

Our study did not clearly show the progress of air-induced xylem embolisms in the branches (Fig. 3d). This might be due to the fact that we used only healthy branches in each tree, and a temporal increase in soil water potential may refill embolized vessels^20^. In this study, the drought-induced damage of accumulation over the long term seems to be apparent in the Huber values, rather than in PLC in branchlets and leaf water potential, as a nonreversible loss of regenerating structure toward tree death. Although the progression of defoliation partially can compensate for the water balance of whole plant under drought, this process reduces carbon gain, which leads to tree death. The values of the midday leaf water potential remained constant (approximately −2 MPa) except in one individual tree with a temporal drop of leaf water potential at midday (Fig. 2b). The large drop in leaf water potential can lead to a decrease in leaf hydraulics (*K*_leaf_)^44^. This finding indicates that leaf water potential drops temporally under severe drought and progresses defoliation. Leaf water potential is then temporally recovered to maintain high leaf water potential at midday as an isohydric behaviour. Thus, this process would be cyclically repeated with the progress of defoliation, and dehydration damage would be accumulated under prolonged drought. From these results, stomatal conductance gradually decreased with defoliation to keep their isohydric behaviour in this species.

Models are important tools for investigating processes and making future projections from individual trees to the entire globe. To implement such an approach, we need to identify the key axes of drought trait trade-offs to link to the probability of species-specific or plant functional type-specific tree mortality under prolonged drought^8^. Our hypothetical framework predicts that tree seedlings, trees growing in the shade and gymnosperms with lower carbohydrate reserves may be more susceptible to lethal drought than adult trees, trees with greater sun exposure, and angiosperms with higher carbohydrate reserves. This prediction corresponds to the published results, supporting the carbon starvation hypothesis via tree-seedling^13^ or gymnosperm^14^ observation, and the hydraulic failure hypothesis via adult-tree^6,9,11,12^ or angiosperm^14^ observation. Among adult trees of different functional types, anisohydric trees have harder wood and fewer parenchyma cells in their sapwood, and they exhibit a greater tolerance to air-induced xylem embolism than do isohydric trees^36^. Therefore, anisohydric woody plants usually show high drought tolerance and can grow in thin-soil sites. However, when prolonged drought occurs, anisohydric trees may be more susceptible because of their low carbohydrate reserves. The long-term resilience of forest trees to the expected increases in heat waves and drought is an important measure that can be used to predict the change of tree species compositions and forest function^3,4^. The obtained hydraulic function–carbon metabolism relationships can be used to predict the pattern of forest degradation during prolonged drought and to select appropriate tree species for reforestation, thereby contributing to the construction of adaptive measures in forest ecosystems facing climatic change.

## Methods

### Study site, climatological measurement and plant materials

The study site (27°07′N, 142°12′E) was located on Ani-jima Island, a territorial island of the Ogasawara (Bonin) Islands in Japan, which are in the northern Pacific Ocean. From 2005 to 2016, the mean air temperature was 23.3 °C and the mean annual precipitation was 1275 mm (Observation by the Japan Meteorological Agency). There are no records of snow falling on these islands.

In the study site, photon flux density (PFD) was measured every 15 min with a quantum sensor (UIZ-PAR-LA, Uijin Co. Ltd, Tokyo, Japan) from 2016 to 2017. Soil water content was also measured at 30 cm depth every 30 min with an ADR soil moisture sensor (SM150; Delta-T Devices Ltd, Cambridge, UK) from 2016 to 2017 (however, data were not obtained from 26 June to 29 November in 2016). These data were automatically stored in data loggers (LR5041 and LR5042; Hioki-Denki Co. Ltd, Nagano, Japan). The seasonal changes in PFD and soil water content are shown in Supplementary Fig. 1.

The soil is of volcanic origin in the study site. The study site was heavily covered by invasive *Lantana camara* L. shrubs, and the plant growth of other tree species was entirely suppressed because of their dense canopy. To promote natural regeneration, an area of approximately 600 m^2^ was cleared of *Lantana* shrubs in December 2013, and the root systems were killed using a herbicide (Roundup; Monsanto Inc., Creve Coeur, MO, USA) in December, 2013. Subsequently, many individual pioneer *Trema orientalis* (L.) Blume trees started to grow. However, severe shading among individual trees was not found, because of the scattered distribution of individual saplings.

When we started this study, the site was entirely open, sun-exposed area. We selected 290 sun-exposed individual *Trema* trees without any symptoms of pathogens in the summer of 2015. In the autumn of 2016, many individual trees set seeds. We selected saplings under sunlit conditions in 2015, and they were already suffering from various levels of defoliation caused by severe drought. In the summer of 2016, the height of the canopy top of the tallest individual tree reached 3.59 m above the ground.

### Plant growth and survival/mortality

We examined the relationships between the RGRs of tree height and stem diameter and the survival/mortality rates along with different defoliation levels. Defoliation is often an important characteristic of trees facing lethal drought^19^. As an index of defoliation levels, we used the Huber value (Huber values, sapwood area/total leaf area). On 17 June 2015, we counted the total number of all leaves, measured the aboveground tree heights, and measured the stem cross-sectional area at 10% of each tree’s height in the 290 individual trees examined. We estimated the total leaf area from the number of leaves and the averaged leaf area for each tree, and we estimated the sapwood area of each tree as the stem cross area multiplied by 0.743. This factor was obtained by cutting ten saplings (0.743 ± 0.055, mean ± 1 SD). The photo-image of cross-sections was analysed with a free software (ImageJ 1.41; U.S. National Institutes of Health, Bethesda, Maryland, USA).

On 25 June 2016, we again measured the tree heights and the stem cross-sectional areas at the same sections of stems (painted in 2015) in the remaining individual trees. The RGR for tree heights in individual trees was calculated as follows:

1 $${\mathrm{RGR}} = \frac{{{\mathrm{ln}}\,({\mathrm{tree}}\,{\mathrm{height}}\,{\mathrm{in}}\,2016)\, - {\mathrm{ln}}\,({\mathrm{tree}}\,{\mathrm{height}}\,{\mathrm{in}}\,2015)}}{{374\,{\mathrm{days}}}}.$$

The RGR of stem cross-sectional area in the individual trees was calculated as follows:

2 $${\mathrm{RGR}} = \frac{{\ln \left( {{\mathrm{stem}}\,{\mathrm{area}}\,{\mathrm{in}}\,2016} \right) - \ln \left( {{\mathrm{stem}}\,{\mathrm{area}}\,{\mathrm{in}}\,2015} \right)}}{{374\,{\mathrm{days}}}}.$$

Mortality over 1 year was examined and categorized as death from drought or unknown.

### Leaf gas exchange, hydraulic function and dark respiration

To investigate the relationships between Huber values and physiological processes along with tree health, including the tree wilting threshold, we selected ten individual trees of 2-year-old with different Huber values and a similar stem size (19.8–24.9 mm in stem diameter at the stem base) out of the 290 individual trees examined (Supplementary Fig. 2).

We measured the photosynthetic rates and stomatal conductance in the just or before fully expanded leaves with a portable open gas exchange system (LI-6400; LI-COR Inc., Lincoln, NE, USA), on 28 July 2015 (2-year-old). The measurements were conducted before noon under the conditions of 400 µmol mol^−1^ CO_2_ in the inlet gas stream and 2000 µmol m^−2^ s^−1^ PFD with red–blue light-emitting diodes. Relative humidity in the out gas stream was adjusted to relative humidity in the ambient air. It is known that the maximum leaf gas exchange rates are found in the leaves before the cessation of leaf expansion in this species^42^. After the leaf gas exchange measurements, we immediately measured the soil and midday leaf water potential values at the noon with a pressure chamber (1505D-EXP; PMS Instrument Company, Albany, OR, USA). The soil-to-leaf hydraulic conductance (*K*_soil-to-leaf_; mmol m^−2^ s^−1^ MPa^−1^) was calculated from the values of soil and midday leaf water potential and transpiration rates, as follows:

3 $$K_{{\mathrm{soil}} \mbox{-} {\mathrm{to}} \mbox{-} {\mathrm{leaf}}} = \frac{E}{{(\psi _{{\mathrm{soil}}} - \psi _{{\mathrm{midday}}})}}$$

where *E* is the transpiration rate per unit leaf area (mmol m^−2 ^s^−1^), and *ψ*_soil_ and *ψ*_midday_ are the soil and midday leaf water potential values (MPa), respectively. The values of *ψ*_soil_ were −0.94 to −1.43 MPa. There is difficulty for exact estimation of *ψ*_soil_ in daytime (it is difficult to entirely eliminate error). In this study, it is assumed that *ψ*_soil_ at daytime is equivalent to daytime leaf water potential of individual trees that transpiration is stopped. The values of *ψ*_soil_ were determined in healthy trees growing next to the ten examined trees (assuming that the soil depth of the roots was similar). To estimate the soil water potential at noon on the measurement day, the measurement leaves were wrapped by polyvinylidene chloride wrap one day before and then the whole individual trees were double covered with plastic bags and thin sheets (with silver colour to avoid sun exposure) to eliminate transpiration^45^. The daytime water potential of the wrapped leaves was determined as daytime *ψ*_soil_.

Using the healthiest branches of individual trees, we examined the hydraulic conductivity and PLC (percent loss of conductivity) of the branches in the ten selected saplings in the summer of 2015. The branches were cut in the early morning and recut under water to avoid the possibility of artificial air-induced embolism. We measured hydraulic conductivity in sections (approximately 10 cm in length) of branches with similar diameters (approximately 6 mm diameter) by gravimetrically adding 5 kPa of hydraulic pressure to the end of the examined twigs from a 50-cm high water bag containing a KCl solution^46^. The maximum vessel length was approximately 8 cm, which was determined by the air-injection method^47^. The PLC was calculated as follows:

4 $${\mathrm{PLC}} = \left( {1 - \frac{{K_{{\mathrm{branch}}}}}{{K_{{\mathrm{max}}}}}} \right)100,$$

where *K*_branch_ is the initial hydraulic conductivity and *K*_max_ is the maximum hydraulic conductivity without air-induced embolisms. All of the hydraulic measurements were performed at a temperature of 25 °C in our laboratory. The other end of the twigs was directly connected to a plastic bottle set on an electronic balance by using a tubing system, and the water flow rates from the twig end were automatically measured. Based on the water flow rates, the sapwood area and the branch length, we calculated the hydraulic conductivity of the branches from each tree.

To evaluate cell activity, dark respiration rates were measured at a temperature of 25 °C in our laboratory in three plant parts: (1) twig at 6 mm in diameter, (2) stem bases (stems between 10% of the tree heights and the part just above the ground) and (3) underground taproots. Each plant part was cut in the field, wrapped with wet paper in a plastic bag and then carried to our laboratory in the summer of 2015. The plant parts were put in a closed plastic box with a fan (0.6, 1.35 or 3.0 L in volume; the size of plastic box was selected according to the volume or length of the samples) covered with thin aluminium foil. We measured the increased rates in CO_2_ concentration in the air in the boxes for 3–10 min with a thin-film capacitance CO_2_ sensor (GM70; Vaisala, Helsinki, Finland). The plant samples were dried at 60 °C for a week and their dry mass was weighed. Respiration rates were calculated based on dry mass.

### Carbohydrate reservation

Non-structural carbon (NSC: starch and soluble sugars) within the sapwood was measured in three plant parts (twigs 6 mm in diameter, stem bases and taproots). The xylem sapwood (without the bark, phloem and pith) from each part was ground to a fine powder and then extracted in 80% ethanol (v/v). The supernatant was extracted via centrifugation and used to quantify the soluble sugar content via the phenol–sulfuric acid method^48^. The starch in the remaining pellets was depolymerized to glucose by the addition of KOH, acetic acid and an amyloglucosidase buffer. After quantifying the glucose extracted via the mutarotase–glucose oxidase method (Glucose C-II test; Wako, Tokyo, Japan), the starch content within the xylem sapwood was measured.

### Tracer experiment for phloem carbon transport

To evaluate the carbon transport capacity in the phloem, we conducted a tracer experiment with a stable isotope of carbon (^13^C) in the field (Supplementary Fig. 3). We selected three individuals with different degrees of health; i.e., a healthy tree (Huber value (sapwood area/total leaf area): 215.5 mm^2^ m^−2^), a defoliated tree (Huber value: 1259.6 mm^2^ m^−2^) and a severely defoliated tree (Huber value: 3101.6 mm^2 ^m^−2^). The mortality rates of these trees over 1 year were approximately 5%, 11% and 43%, respectively (see Fig. 1c). On 4 July 2016, we collected part of the phloem of four plant parts as a control: the base of the twig that is planned for use in ^13^C labelling (called the twig); the part of the stem above the labelled twig (called the top stem); the part of the stem between the labelled twig and the stem base (called the middle stem); and the stem base in each tree. Because the severely defoliated tree had only one healthy twig and the top stem had died back, we were not able to collect the top stem for this plant. The next day, we conducted the labelling experiment. We wrapped the healthiest branch overall with a plastic bag and periodically added air with the labelled ^13^CO_2_ into the plastic bag during the morning (8:20–10:45 h) (Supplementary Fig. 3). The size of the plastic bag was approximately 20 L for the defoliated and was severely defoliated trees, and was approximately 60 L for the healthy tree. In total, the mol fractions (input ^13^CO_2_ air/air) were approximately 33.6 mmol mol^−1^ for the severely defoliated tree, 8.4 mmol mol^−1^ for the defoliated tree and 11.2 mmol mol^−1^ for the healthy tree depending on the variations in photosynthetic activity. The ^13^C labelling experiment was conducted on a relatively cloudy day with frequent exposure to sunshine. Because drops of dew were detected inside the plastic bags, stomatal opening was maintained during the labelling. On the following days after labelling (1, 2 and 5 days later), we continuously collected a part of the phloem at the four/three portions of the plants with fresh razor blades. The collected phloem was dried at 60 °C and ground to a fine powder in our laboratory. The abundance of ^13^C in each sample was evaluated based on an index, the atom% of ^13^C index, as follows:

5 $${\mathrm{atom}}{{\%} \, {\mathrm{of}}}\,^{13}{\mathrm{C}} = \left( {\frac{{{\mathrm{amount}}\,{\mathrm{of}}\,^{13}{\mathrm{C}}}}{{{\mathrm{total}}\,{\mathrm{amounts}}\,{\mathrm{of}}\,^{13}{\mathrm{C}}\,{\mathrm{and}}\,^{12}{\mathrm{C}}}}} \right)100.$$

The atom% of ^13^C was measured with a Flash EA1112-DELTA V PLUS ConFlo III System (ThermoFisher Scientific Inc., Waltham, MA, USA) by SI Science Co. Ltd (Saitama, Japan). The values for atom% in the control (before ^13^C labelling) were 1.071–1.073, and they were independent of the sampled plant parts sampled and individual trees. After labelling, the atom% values in the bases of the labelled twig reached 1.084–1.101 one day after labelling.

To evaluate the carbon transport capacity in the phloem, we calculated the increase in ^13^C (atom% of ^13^C in the labelling−atom% of ^13^C in the control) in each plant part on each day of sampling. These values were then divided by the value in the labelled twig one day after labelling in each tree to standardize the variations in the amounts of ^13^C absorbed among the individual trees.

### Statistics

Statistical analyses were conducted with R Ver. 3.3.1 (R Development Core Team, R Foundation for Statistical Computing, Vienna, Austria). For the relationships between the measured plant traits and Huber values in Fig. 3, the linear and quadratic relationship fits were compared via ANOVA (F-test; Supplementary Table 1), and the regression lines were drawn depending on the significant levels (Supplementary Table 2). The Huber value values (mm^2^ m^−2^) were log-transformed. A PCA was conducted, using nine plant traits and the ten individuals in Fig. 3. Pearson’s correlations for each pair of traits were examined among the nine plant traits used in the PCA (Supplementary Table 3). The values obtained for each parameter are standardized as follows: (obtained value−mean value)/standard deviation.

## Supplementary information

Supplementary Information

## Data Availability

All raw data in the current study are available from the Dryad Digital Repository^49^ at 10.5061/dryad.8j60c45.
