# Supplementary material for: Initial hydraulic failure followed by late-stage carbon starvation leads to drought-induced death in the tree Trema orientalis
Source: Commun Biol. 2019 Jan 7;2:8. doi: 10.1038/s42003-018-0256-7 (PMC6323055; doi:10.1038/s42003-018-0256-7)
Supplement: Supplementary file 1 — Supplementary Information [file 42003_2018_256_MOESM1_ESM.pdf]

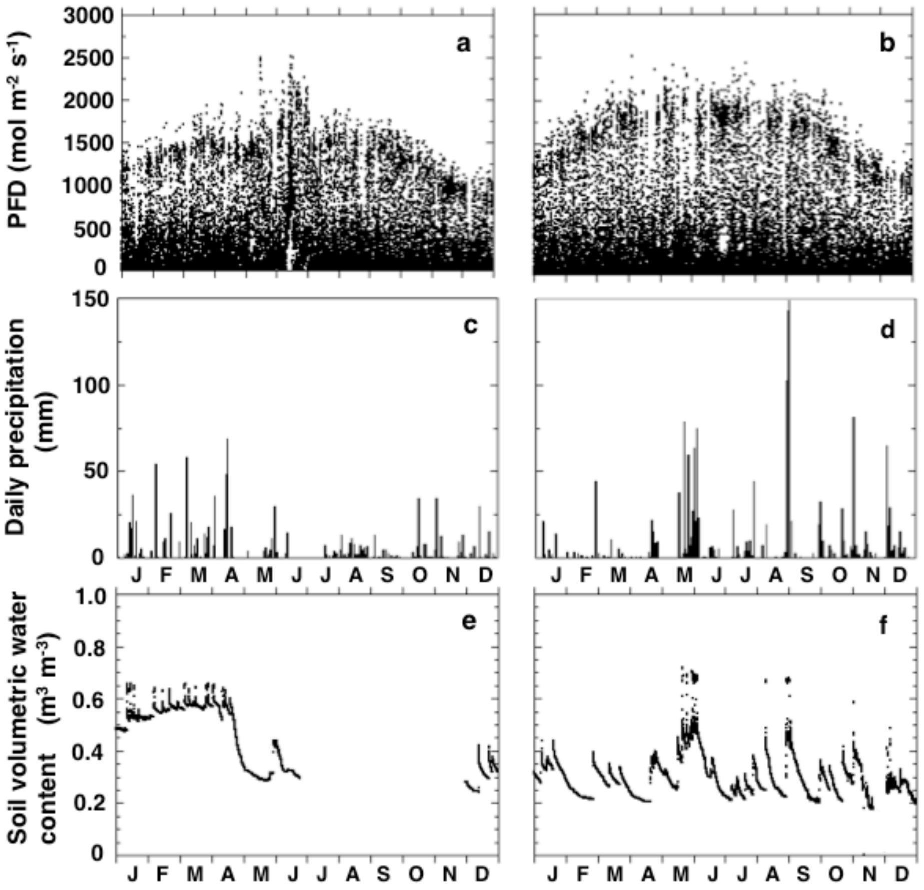

Supplementary Figure 1. The seasonal changes in photon flux density (PFD), daily precipitation and soil volumetric water contents at 30 cm depth in 2016 (**a**, **c, e**) and 2017 (**b**, **d, f**). The annual precipitation was 1021.0 mm in 2016, and 1657.5 mm in 2017. Data of daily precipitation are from the Japan Meteorological Agency. The values of PFD were obtained every 15 min. Data for soil volumetric water contents were not obtained during the period from 26 June to 29 November in 2016.

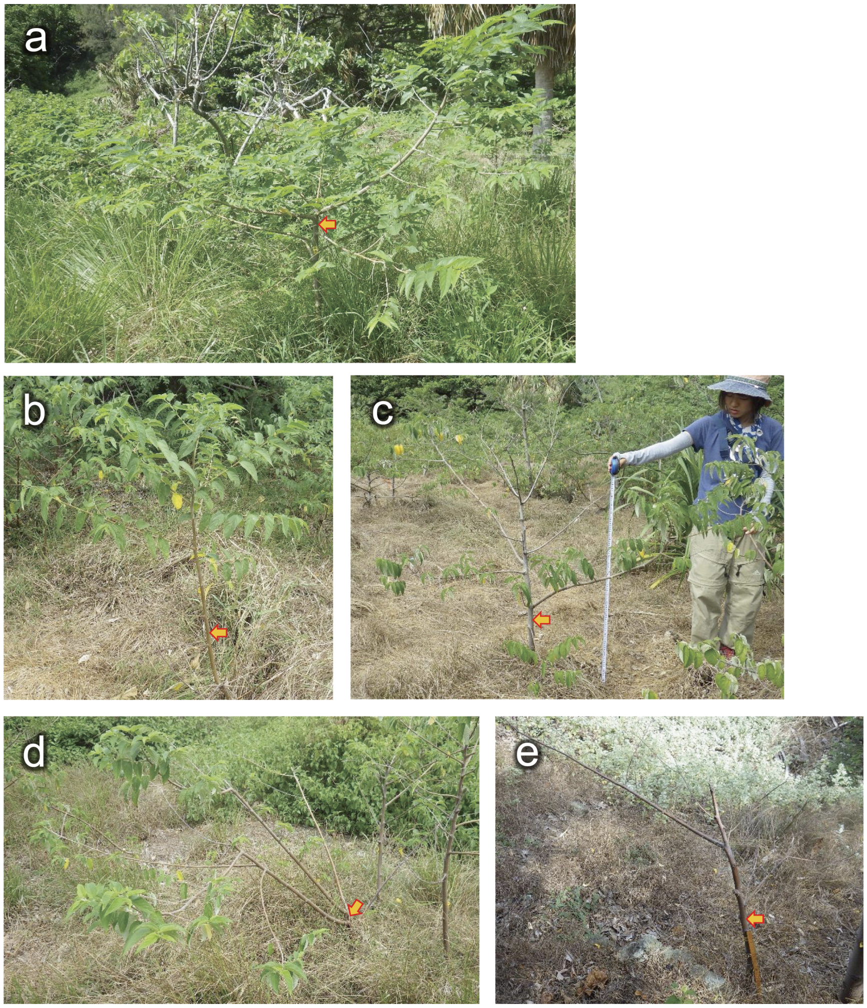

Supplementary Figure 2. Photographs of individual trees, shown by arrows, with different Huber values (HVs: sapwood area/total leaf area). **a** 248.13 mm^2 m^{-2} (log(HV) = 2.39), **b** 265.70 mm^2 m^{-2} (log(HV) = 2.42) mm^2 m^{-2}, **c** 1183.1 mm^2 m^{-2} (log(HV) = 3.07) and **d** 1951.4 mm^2 m^{-2} (log(HV) = 3.29) in HVs. **e** The HV was unable to be measured, because of the lack of leaves. The arrow shows the individual tree in each panel.

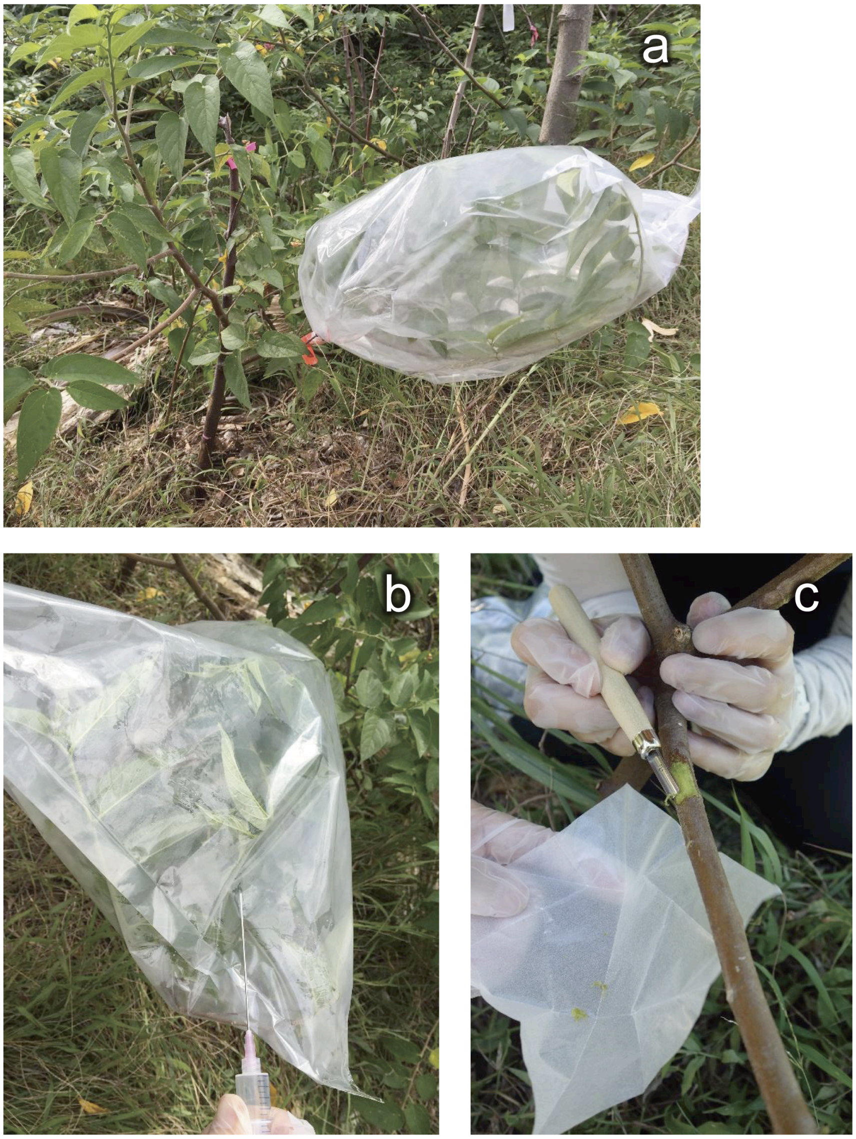

Supplementary Figure 3. Photographs of the ^{13}C labeling experiment in the field. **a** The healthy branch was sealed in a plastic bag for the ^{13}C labeling experiments. See the supplementary methods for details. **b** The injection of $^{13}CO_2$ into the hermetically sealed space. **c** Collecting a sample of phloem with a blade.

Supplementary Table 1. The values of adjusted r^2, F, and P in the relationships between the plant traits and HVs in Figure 3.

Plant traits	unit	Adjusted r^2 in linear regression line	Adjusted r^2 in quadric regression line	F value	P value	Selected regression lines
Photosynthetic rate	μmol m^{-2} s^{-1}	0.62	0.60	0.68	0.44	Linear
Stomatal conductance	mol m^{-2} s^{-1}	0.45	0.38	0.004	0.95	Linear
Leaf water potential at midday	MPa	-0.12	-0.14	0.85	0.39	Linear
$K_{soil\text{-}to\text{-}leaf}$	mmol m^{-2} s^{-1} MPa^{-1}	0.26	0.16	0.04	0.84	Linear
K_{branch}	kg m^{-1} s^{-1} MPa^{-1}	0.25	0.17	0.19	0.68	Linear
PLC (Percent loss of conductivity)	%	0.09	-0.03	0.11	0.75	Linear
Respiration rate in branch	μmol g^{-1} s^{-1}	0.37	0.29	0.01	0.94	Linear
Respiration rate in stem base	μmol g^{-1} s^{-1}	0.39	0.30	0.03	0.88	Linear
Respiration rate in tap-root	μmol g^{-1} s^{-1}	-0.09	-0.18	0.37	0.56	Linear
Soluble sugar contents in branch	g g^{-1}	-0.09	-0.05	1.32	0.29	Linear
Soluble sugar contents in stem base	g g^{-1}	0.28	0.52	4.98	0.06	Linear
Soluble sugar contents in tap-root	g g^{-1}	0.39	0.44	1.70	0.23	Linear
Starch contents in branch	g g^{-1}	-0.01	-0.15	0.004	0.95	Linear
Starch contents in stem base	g g^{-1}	-0.05	0.48	9.08	0.02	**Quadric**
Starch contents in tap-root	g g^{-1}	-0.09	0.01	1.84	0.22	Linear
NSC in branch	g g^{-1}	-0.06	-0.19	0.12	0.74	Linear
NSC in stem base	g g^{-1}	-0.12	0.57	13.98	0.01	**Quadric**
NSC in tap-root	g g^{-1}	-0.10	0.11	2.88	0.13	Linear

Supplementary Table 2. The values of estimated coefficients, standard error, *T* values and *P* values for the selected regression lines in the relationships between the plant traits and HVs in Figure 3.

Plant traits	unit	Selected regression	Intercept and variables	Estimated coefficients	Standard error	*T* value	*P* value
Photosynthetic rate	μmol m^{-2} s^{-1}	Liner	Intercept	26.174	5.479	4.778	**
			x	7.272	1.854	-3.922	**
Stomatal conductance	mol m^{-2} s^{-1}	Liner	Intercept	0.320	0.088	3.635	**
			x	-0.087	0.030	-2.914	*
Leaf water potential at the midday	MPa	Liner	Intercept	-1.821	1.890	-0.964	n.s.
			x	-0.130	0.639	-0.203	n.s.
$K_{soil\text{-}to\text{-}leaf}$	mmol m^{-2} s^{-1} MPa^{-1}	Liner	Intercept	4.721	1.896	2.490	*
			x	-1.307	0.641	-2.038	marginal (*P* = 0.0759)
K_{branch}	kg m^{-1} s^{-1} MPa^{-1}	Liner	Intercept	14.118	4.562	3.095	*
			x	-3.101	1.544	-2.009	marginal (*P* = 0.0794)
PLC (Percent loss of conductivity)	%	Liner	Intercept	-36.520	40.760	-0.896	n.s.
			x	18.770	13.790	1.361	n.s.
Respiration rate in branch	μmol g^{-1} s^{-1}	Liner	Intercept	0.018	0.005	3.406	**
			x	-0.005	0.002	-2.526	*
Respiration rate in stem base	μmol g^{-1} s^{-1}	Liner	Intercept	0.011	0.003	3.747	**
			x	-0.003	0.001	-2.591	*
Respiration rate in tap-root	μmol g^{-1} s^{-1}	Liner	Intercept	0.001	0.003	0.315	n.s.
			x	0.001	0.001	0.534	n.s.
Soluble sugar contents in branch	g g^{-1}	Liner	Intercept	0.018	0.011	0.152	n.s.
			x	0.002	0.004	0.486	n.s.
Soluble sugar contents in stem base	g g^{-1}	Liner	Intercept	-0.011	0.022	-0.505	n.s.
			x	0.015	0.007	2.117	marginal (*P* = 0.0671)
Soluble sugar contents in tap-root	g g^{-1}	Liner	Intercept	-0.033	0.036	-0.905	n.s.
			x	0.032	0.012	2.610	*
Starch contents in branch	g g^{-1}	Liner	Intercept	0.064	0.037	1.744	n.s.
			x	-0.012	0.012	-0.977	n.s.

Starch contents in stem base	$g\ g^{-1}$	Quadric	Intercept	-0.832	0.304	-2.734	*
			x	0.617	0.209	2.949	*
			x^2	-0.107	0.036	-3.013	*
Starch contents in tap-root	$g\ g^{-1}$	Liner	Intercept	0.128	0.093	1.376	n.s.
			x	-0.015	0.031	-0.484	n.s.
NSC in branch	$g\ g^{-1}$	Liner	Intercept	0.082	0.042	1.951	n.s.
			x	-0.010	0.014	-0.719	n.s.
NSC in stem base	$g\ g^{-1}$	Quadric	Intercept	-1.198	0.341	-3.515	**
			x	0.878	0.234	3.746	**
			x^2	-0.149	0.040	-3.739	**
NSC in tap-root	$g\ g^{-1}$	Liner	Intercept	0.095	0.108	0.883	n.s.
			x	0.017	0.037	0.452	n.s.

** $P < 0.01$, * $P < 0.05$, "marginal" $0.05 \leq P < 0.08$, n.s. $P > 0.08$

Supplementary Table 3. Pearson's correlations for each pair of plant traits used in the PCA shown in Figure 5a. Correlation coefficients (*r*) are given in the lower left section, and the positive (+) or negative (-) relationship for each pair is given in the upper right section of the matrix. See the legend of Figure 5 for abbreviations.

	*A*max	*G*s	*K*branch	*K*soil-leaf	*R*branch	*R*stem	*ST*stem	*SS*stem	*NSC*stem
*A*max		**(+)		*(+)		*(+)			
*G*s	0.780			*(+)		***(+)		*(-)	
*K*branch	0.224	0.403						*(-)	
*K*soil-leaf	0.685	0.683	0.293						
*R*branch	0.543	0.191	0.424	0.401					
*R*stem	0.730	0.845	0.265	0.267	0.138				
*ST*stem	0.109	0.238	-0.021	-0.152	-0.140	0.426			***(+)
*SS*stem	-0.523	-0.688	-0.701	-0.400	-0.218	-0.570	0.208		
*NSC*stem	-0.143	-0.111	-0.327	-0.300	-0.210	0.093	0.902	0.611	
